# Assessment of Drug Delivery Kinetics to Epidermal Targets *In Vivo*

**DOI:** 10.1208/s12248-021-00571-3

**Published:** 2021-03-29

**Authors:** M. Hoppel, M. A. M. Tabosa, A. L. Bunge, M. B. Delgado-Charro, R. H. Guy

**Affiliations:** 1 Department of Pharmacy & Pharmacology, University of Bath, Claverton Down, Bath, BA2 4LZ UK; 2 Department of Chemical and Biological Engineering, Colorado School of Mines, Golden, Colorado 80401 USA

**Keywords:** dermal pharmacokinetics, *in vitro* skin permeation, stratum corneum sampling, topical drug delivery, transdermal delivery systems

## Abstract

It has proven challenging to quantify ‘drug input’ from a formulation to the viable skin because the epidermal and dermal targets of topically applied drugs are difficult, if not impossible, to access *in vivo*. Defining the drug input function to the viable skin with a straightforward and practical experimental approach would enable a key component of dermal pharmacokinetics to be characterised. It has been hypothesised that measuring drug uptake into and clearance from the stratum corneum (SC) by tape-stripping allows estimation of a topical drug’s input function into the viable tissue. This study aimed to test this idea by determining the input of nicotine and lidocaine into the viable skin, following the application of commercialised transdermal patches to healthy human volunteers. The known input rates of these delivery systems were used to validate and assess the results from the tape-stripping protocol. The drug input rates from *in vivo* tape-stripping agreed well with the claimed delivery rates of the patches. The experimental approach was then used to determine the input of lidocaine from a marketed cream, a typical topical product for which the amount of drug absorbed has not been well-characterised. A significantly higher delivery of lidocaine from the cream than from the patch was found. The different input rates between drugs and formulations *in vivo* were confirmed qualitatively and quantitatively *in vitro* in conventional diffusion cells using dermatomed abdominal pig skin.

**Supplementary Information:**

The online version contains supplementary material available at 10.1208/s12248-021-00571-3.

## INTRODUCTION

Modelling ‘drug input’ into the skin from topical formulations is challenging because the most common sites of action of these products, the viable epidermal and upper dermal compartments, are experimentally difficult to access *in vivo*. To predict the local (epi)dermal pharmacokinetics and target tissue concentrations, considerable effort has been made to model mathematically the drug transfer from its vehicle into the stratum corneum (SC), its subsequent diffusion across this barrier and its transfer into the viable epidermal/dermal tissue (1). Validation of such predictions requires experimental determinations of the drug input function to the viable skin from the applied formulation. While local drug concentrations in the skin can be measured using spectroscopic techniques (e.g. Raman/IR) or by microdialysis/microperfusion (2–5), these approaches are limited by sensitivity issues, on the one hand, and by considerable technical demands, on the other.

An alternative method to determine the concentration of an active entity in the viable epidermis is based on the measurement of drug uptake from a formulation into and elimination of the drug from the SC by tape-stripping, as described recently for acyclovir (6).

The idea behind this approach was first indicated and justified through mathematical modelling (7), and further illustrated in later publications on diclofenac (8) and metronidazole (9). Specifically, the clearance phase, where the drug diffuses from the SC into the deeper layers of the viable skin below, provides valuable information about a topical drug’s input function into the viable tissue (6–9). It follows that the mass of drug in the viable tissue depends on the flux of drug from the SC and the rate of its subsequent transfer to the pre-systemic blood compartment. Therefore, measurement of drug clearance from the SC provides information about topical drug input kinetics into the epidermis/dermis (7).

Hence, for drugs with their site of action in the viable epidermis, the SC sampling methodology, as recently described (6–9), is targeted at eliciting information directly pertinent to the assessment of topical bioavailability (and by extrapolation, eventually, to bio(in)equivalence between different formulations). As such, the new approach to the interpretation of SC sampling data responds directly to an early and still-voiced criticism of tape-stripping that it cannot provide a useful metric related to the rate and extent of drug delivery to sites of action that are found elsewhere in the skin (such as the viable epidermis).

Of course, the ‘input’ function only provides part of the information needed to estimate a drug’s concentration in the sub-SC compartment and that requires knowledge of the local clearance too (and may be even more complicated for drugs subject to epidermal metabolism). However, there are models and associated algorithms for estimating ‘dermal clearance’ by the local microcirculation (10,11) and these can be used, together with the delivery rate of drug from SC obtained from tape-stripping experiments, to calculate a concentration in a viable skin compartment—the C* value as described by Higuchi *et al*. many years ago (12–16).

The aim of this study is first to validate the *in vivo* SC sampling method using transdermal drug delivery systems for nicotine and lidocaine. Although drugs administered from conventional patches are not typically designed to target structures within the skin, the active entities must pass through the epidermis/dermis en route to the systemic compartment (17). For all approved transdermal patches, the labelling specifically includes information on the drug input rate at steady-state (in amount per unit time) and permits, therefore, an investigation of whether these values can be duplicated using the SC tape-stripping protocol in healthy human volunteers (7). Having established a suitable protocol, the input kinetics of lidocaine from a marketed cream formulation (for which the clinical indication is different from that of the patch (18)) into the viable skin are then assessed. The cream represents a typical topical product, of course, that undergoes a significant ‘metamorphosis’ (19) during and immediately after its application to the skin, complicating thereby the drug uptake and clearance kinetics into and out of the SC. Finally, to complement the method validation, the *in vivo* experiments are replicated, as far as is possible, in more conventional *in vitro* permeation tests using abdominal pig skin as a recognised model membrane (20) for the human counterpart.

## MATERIALS AND METHODS

### Materials

Disodium hydrogen phosphate was purchased from Acros Organics (Geel, Belgium), potassium dihydrogen phosphate from Fisher Scientific (Loughborough, UK), acetonitrile and methanol from VWR Chemicals (Lutterworth, UK). Other solvents, HPLC reagents, lidocaine and (−)-nicotine were obtained from Sigma-Aldrich (Gillingham, UK). Nicotinell® 7 mg/24 h patches (Novartis Consumer Health, Camberley, UK) were bought from Boots UK Limited (Nottingham, UK). Versatis® 5 mg medicated plaster was from AAH Pharmaceuticals Ltd (Coventry, UK). LMX4 (Lidocaine 4% w/w cream) was purchased from HI Weldrick Ltd (Doncaster, UK).

### Methods

#### *In Vivo* Experiments

##### Subjects

Eighteen healthy volunteers with no history of dermatological disease participated in the study. Six volunteers were enrolled for each treatment: three males and three females (age range 25–34 years) for the nicotine patch; two males and four females (age range 25–34 years) for the lidocaine patch; three males and three females (age range 25–28 years) for the lidocaine cream. The different protocols were approved by the Research Ethics Approval Committee for Health (REACH) of the University of Bath: REACH 15/16 112 (nicotine) and REACH 16/17 006 (lidocaine). Informed consent was obtained from each subject.

##### SC Sampling

This study followed a published SC sampling method [7] with a few modifications. One hour before application, the volunteers’ forearms were cleaned with a mild soap solution (Cussons Carex complete antibacterial handwash, PZ Cussons, Manchester, UK), rinsed thoroughly with warm water and dried. The same formulation was then applied to both forearms providing duplicate measurements in each volunteer, as described in further detail below. In the earlier study (7), the number of tape-strips required to collect most of the SC was determined by periodic measurements of transepidermal water loss (TEWL) of each treated site; in contrast, here, the number of tape-strips applied to all treated sites for each subject was the same and was based on the average number of tapes required for TEWL (measured with an AquaFlux AF102, Biox Systems Ltd., London, UK) to surpass 60 g m^−2^ h^−1^ (or a maximum of 30 tape-strips) for the two untreated (blank) sites on that subject. This was a practical necessity for the nicotine study because the occlusive nature of these patches interfered with the TEWL readings. The lidocaine study, which was conducted after the nicotine experiments, followed the same procedure, except that the TEWL was determined immediately before and after the tape-stripping of each treated site. TEWL measurements of the untreated site were performed before tape-stripping, then occasionally as tape-stripping proceeded, and after 30 strips if the TEWL did not exceed 60 g m^−2^ h^−1^ first. Typically, 20–30 tape-strips were required.

The amount of SC on the tapes removed from treated and untreated sites was determined gravimetrically using a microbalance (SE-2F, precision 0.1 μg; Sartorius AG, Göttingen, Germany) after being discharged of static electricity (R50 discharging bar with ES50 power supply from Eltex Electrostatic GmbH, Weil am Rhein, Germany). From the area tape-stripped and the known density (~ 1 g cm^−3^) of the SC (21), the mass on each tape was converted to the corresponding thickness removed.

##### Nicotine

**Fig. 1:**
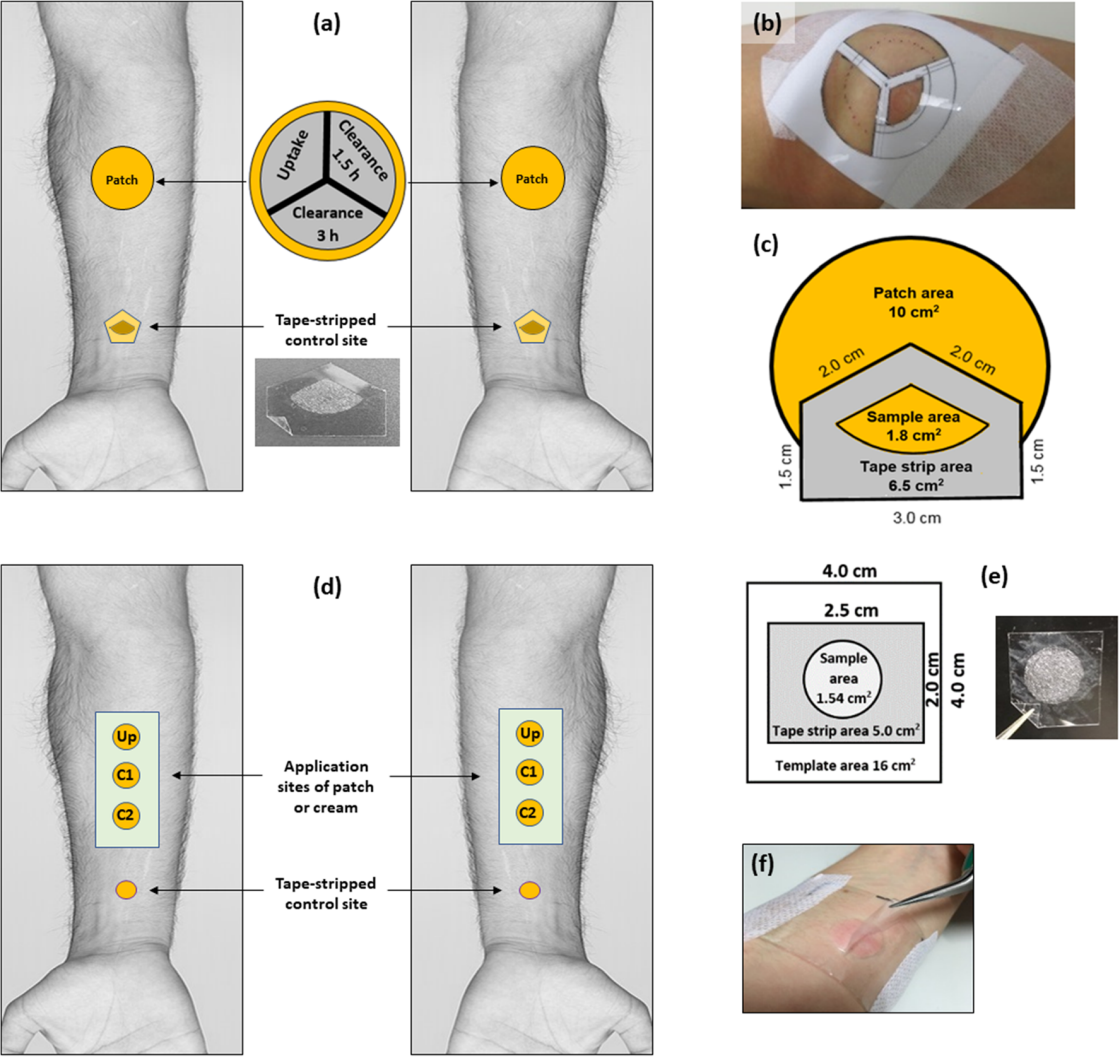
Pictorial representation of the *in vivo* SC sampling experiments for nicotine (panels **a**, **b** and **c**) and lidocaine (panels **d**, **e** and **f**). See text for details

One Nicotinell® 7 mg/24 h patch was applied on each forearm of the volunteers (*n* = 6). After 2 h, both patches were removed, leaving no detectable residue on the skin, and the application sites were demarcated into three equal areas of 1.8 cm^2^ using a template cut from Scotch Book tape 845 (3M, St. Paul, MN, USA) (Fig. 1a). One area was tape-stripped immediately (‘uptake’), a second at 1.5 h after patch removal (‘1.5-h clearance’) and the third at 3-h post-patch removal (‘3-h clearance’). Pieces of the same adhesive tape (that were larger than the stripped area) were used to remove the SC with a standardised procedure (7). The template ensured that the application of sequential tape-strips was made on exactly the same location and was designed so that areas of treated skin, which had not yet been stripped, remained unoccluded (Fig. 1b). Because patch adhesion was less consistent at the very edges of the patch, the template ensured that tape-stripping here was avoided (Fig. 1c). All tape-stripping was performed by a single investigator.

Having collected SC at the ‘uptake’ site immediately after patch removal, the two ‘clearance’ sites were protected with a rectangular frame (5.5 cm × 5.5 cm) made of self-stick adhesive pads (Dr. Scholl Pressure Point Foam Padding, Slough, UK) and covered with a non-occlusive mesh (ultra stiff plastic canvas, 7 mesh, Darice®, OH, USA) that was held in place with Mefix (Molnlycke, Oldham, UK). At 1.5 h after patch removal, the dressing was removed and SC from this first ‘clearance’ site was then collected in exactly the same manner as that from the ‘uptake’ area. At 3 h, the procedure was repeated at the second ‘clearance’ site.

##### Lidocaine

To a first group of six volunteers, a Versatis® 5% medicated plaster (or ‘patch’), equivalent to 5 mg cm^−2^ of lidocaine, was applied to three sites on each forearm (Fig. 1d). Patches were worn overnight for 12 h and were then removed. The treated skin areas were subsequently cleaned quickly with a 70% isopropyl alcohol wipe (Sterets®, Molnlycke) to completely remove any residual patch material on the skin. In a second cohort of 6 volunteers, both forearms were treated (at a dose consistent with the Summary of Product Characteristics (22)) with 150 mg cm^−2^ of LMX4 Lidocaine 4% w/w cream, equal to 6 mg cm^−2^ of drug, for 1 h. Removal of residual formulation on the skin involved first dry-wiping with absorbent tissue before cleaning with isopropyl alcohol as for the patch. The selected application times of the two formulations reflected the dosing instructions in the corresponding patient information leaflets.

Demarcation of 1.5 cm^2^ areas of each application site was then made using a template cut from Scotch Book tape 845 (3M) (Fig. 1e). The template ensured that SC collected during the repeated, sequential tape-stripping all originated from exactly the same location. The tape-strips used to remove the SC were much larger than the treated area of SC (Fig. 1e and f). On each arm, for both formulations, one site was tape-stripped immediately (‘uptake’ (Up)), a second at 4 h after patch removal (‘4-h clearance’ (C1)) and the third 8 h post-patch removal (‘8-h clearance’ (C2)) (Fig. 1d). Between formulation removal of the two ‘clearance’ sites (C1 and C2), the skin was protected with a light, non-occlusive gauze (Boots, Nottingham, UK). Again, all tape-stripping was performed by a single investigator.

##### Drug Extraction from SC Tape-Strips

Nicotine was extracted from the SC removed on each tape-strip by shaking overnight with 1.5 mL of the mobile phase used for the subsequent HPLC analysis: phosphate buffer 10 mM, pH 7.4/acetonitrile/methanol, 30:35:35 v/v. The efficiency of nicotine extraction was determined using tape-strips with adhering SC that had been ‘spiked’ with a known amount of the drug; the mean recovery (± SD) was 96.8 ± 3.5% (*n* = 3).

Lidocaine was extracted from SC on groups of 2 to 4 tape-strips, which were rolled, placed into small vials with 3 mL of the same HPLC mobile phase used for nicotine extraction and subjected to 20 min of ultra-sonication (Clifton Ultrasonic Water Bath, Nickel-Electro Ltd., Weston-super-Mare, UK). The efficiency of lidocaine extraction was assessed in the same way as that used for lidocaine; the mean recovery (± SD) was 97.9 ± 2.0% (*n* = 3).

#### *In vitro* Experiments

Abdominal pig skin was obtained post-sacrifice without having been exposed to the normal high-temperature cleaning procedure. Skin was dermatomed (Zimmer®, Warsaw, IN, USA) to a nominal thickness of 750 μm and stored frozen. Before use, the skin was thawed, and any visible hairs were trimmed with scissors.

##### Nicotine

The nicotine patch was cut into circular discs (diameter of 1.4 cm, surface area 1.54 cm^2^) and adhered to the external side of the skin (pressure having been applied with a custom-made roller passed 10 times in two directions to ensure complete adhesion). The skin was mounted in a glass Franz diffusion cell having an internal diameter of 1.4 cm (PermeGear, Inc., Bethlehem, PA, USA). The receptor chamber had been filled with phosphate-buffered saline solution (10 mM, pH 7.4, 7.1 mL). The receptor solution was stirred magnetically at a constant speed of 500 rpm and the temperature was maintained at 37 ± 1 °C by circulating warmed water through a jacket surrounding the cell.

The cumulative delivery of nicotine into the receptor solution was determined following a single 5-h application of the nicotine patch. Aliquots (1 mL) were withdrawn at 1, 2, 3.5 and 5 h, then immediately replaced with the same volume of fresh receptor solution. Samples were filtered (Cronus syringe filter, nylon, 4 mm, 0.45 μm, LabHut, Gloucester, UK) and the concentration of nicotine in the samples was quantified by HPLC as described below. Six replicates were performed for each experiment.

The amount of drug permeated in each sampling interval was calculated from its concentration in the receptor solution and the volume of receptor chamber. The cumulative amount of drug permeated as a function of time was calculated.

##### Lidocaine

The lidocaine patch was cut into circular discs (diameter of 1.4 cm, surface area 1.54 cm^2^) and firmly applied to the external side of the skin (as for nicotine). The cream was applied in the same amount as *in vivo* (150 mg cm^−2^ of cream) using a cotton bud. The skin was mounted in Franz diffusion cells operated as for nicotine except that the temperature in these experiments was controlled by placing the cells in an oven at 32°C. The cumulative amount of lidocaine delivered post-drug application was determined by sampling (with replacement) of the receptor solution at 2, 4, 6, 8, 10 and 12 h for the patch, and at 1, 3, 5, 7 and 9 h for the cream; seven replicates were performed for each formulation. All samples were filtered (Cronus syringe filter, nylon, 4 mm, 0.45 μm, LabHut, UK) and the concentration of lidocaine in the samples was quantified by HPLC as described below. The steady-state flux and lag time (intercept of the time axis) were estimated from the linear gradient of the cumulative amount of drug penetrated *versus* time profile using measurements at times that exceeded 2.4 × the lag time for each cell (23) or from the last sampling interval (for some experiments with the patches) when 2.4 × the lag time exceeded 10 h.

#### Analysis and Interpretation of *In Vivo* Skin Uptake and Penetration Data

The amount of drug in the SC was measured immediately after the applied formulation (patch or cream) is removed, the so-called uptake, and then after two periods of ‘clearance’. In a first approach, the SC is considered to be a well-stirred compartment, from which drug is eliminated with 1st-order kinetics described by a rate constant β. The ‘clearance’ of drug from the SC can therefore be described as follows:

1 $$ {\mathrm{dM}}_{\mathrm{SC}}/\mathrm{dt}=-\upbeta \times {\mathrm{M}}_{\mathrm{SC}} $$

where M_SC_ is the amount of drug in the SC at (‘clearance’) time *t* after formulation removal. The relevant, initial boundary condition is that, at *t* = 0 (i.e. when uptake is finished), M_SC_ = M_UP_, where M_UP_ is the drug amount in the SC at the end of the uptake period. Equation (1) may then be solved:

2 $$ {\mathrm{M}}_{\mathrm{SC}}={\mathrm{M}}_{\mathrm{UP}}\times {\mathrm{e}}^{-\upbeta \mathrm{t}} $$

and

3 $$ \ln\ {\mathrm{M}}_{\mathrm{SC}}=\ln\ {\mathrm{M}}_{\mathrm{UP}}-\upbeta \times \mathrm{t} $$

It follows that the *in vivo* experiments provide measurements of M_SC_ at three ‘clearance’ times, i.e. 0, 1.5 and 3 h following application of the nicotine patch, and 0, 4 and 8 h following administration of both the lidocaine patch and cream. Linear regression of the geometric mean values of the duplicate measurements at uptake and two clearance times yields values of M_UP_ and β from the intercept and from the intercept and slope; these parameters can then be used to ‘estimate’ for each subject the input rate (*R*_1_) of drug from the SC into the underlying viable skin compartment at the moment the ‘uptake’ finished, i.e.

4 $$ {R}_1=-{\left({\mathrm{dM}}_{\mathrm{SC}}/\mathrm{dt}\right)}_{\mathrm{t}=0}=\upbeta \times {\mathrm{M}}_{\mathrm{UP}} $$

Assuming β is constant over the entire time of clearance. The input rate can also be estimated for just the shorter clearance interval (*R*_1,CL1_) as

5 $$ {R}_{1,\mathrm{CL}1}={\upbeta}_{\mathrm{CL}1}\times {\mathrm{M}}_{\mathrm{UP}}=\left[\ln \left({\mathrm{M}}_{\mathrm{UP}}/{\mathrm{M}}_{\mathrm{CL}}\right)/\Delta  \mathrm{t}\right]\times {\mathrm{M}}_{\mathrm{UP}} $$

where M_UP_ and M_CL_ are the geometric mean values of the duplicate measurements determined respectively at uptake and at clearance for ∆t = 1.5 h for nicotine and 4 h for lidocaine. If β is not the same for both the shorter and longer clearance times, then we can expect that R_1,CL1_ will be a better estimate of the input rate to the viable tissue before the drug product is removed from the skin surface.

A second approach that has been reported in the literature (6,8,9) estimates the input rate (*R*_2_) from the difference between the amounts of drug in the SC after ‘uptake’ (M_UP_) and after a period of ‘clearance’ (M_CL_) divided by the time elapsed between the two measurements (∆t):

6 $$ {R}_2=\left({\mathrm{M}}_{\mathrm{UP}}-{\mathrm{M}}_{\mathrm{CL}}\right)/\Delta  \mathrm{t} $$

Calculation of *R*_2_ for the shorter clearance time should provide the closest comparison to the input rate before the drug product is removed and to the estimated input rate by the first approach (i.e. *R*_1_ if β is constant, and *R*_1,CL1_ if β is not the same constant for the shorter and longer intervals). For the first clearance interval, *R*_2_ depends on the fraction of mass remaining in the SC as follows:

7 $$ {R}_2={\mathrm{M}}_{\mathrm{UP}}\ \left(1-{\mathrm{M}}_{\mathrm{CL}1}/{\mathrm{M}}_{\mathrm{UP}}\right)/\Delta  \mathrm{t} $$

It follows that the calculated values of *R*_2_ and *R*_1,CL1_ (Eq. 5) will be equivalent whenever the fraction that cleared from the SC (i.e., 1 − M_CL1_/M_UP_) is small. The clearance rate from the SC slows as the drug amount in the SC decreases. As a result, even if β does not change, the average flux over the interval (represented by *R*_2_) decreases, whereas the clearance rate represented by *R*_1,CL1_ does not because it is estimated from the drug amount in the SC when clearance begins.

#### HPLC Analysis

Nicotine was quantified by HPLC (LC-2010, AHT, Shimadzu, Milton Keynes, UK) with UV detection (206 nm) (24,25) using a mixture of phosphate buffer 10 mM, pH 7.4: acetonitrile:methanol (30:35:35 v/v) as the mobile phase. Samples were filtered (Cronus syringe filter, nylon, 4 mm, 0.45 μm) prior to analysis and run on a C18 column (Kya Tech, London, UK) with a precolumn (Phenomenex, Macclesfield, UK). The flow rate was 1 mL min^−1^ and the column oven temperature was 25 °C. Runtime was 4 min and the nicotine retention time was ~ 3.1 min. A calibration curve was established between 10 and 0.16 μg mL^−1^ with *R*^2^ = 0.999. The limits of detection (LOD) and quantification (LOQ) were 0.05 μg mL^−1^ and 0.17 μg mL^−1^, respectively, and correspond to 0.04 μg cm^−2^ and 0.14 μg cm^−2^ of drug per tape-strip.

Lidocaine was also quantified by HPLC (Summit, Dionex, Swindon UK) with UV detection (240 nm) using a mixture of phosphate buffer 10 mM, pH 7.4:methanol (30:70 v/v) as the mobile phase. All samples were filtered (Cronus syringe filter, nylon, 4 mm, 0.45 μm) prior to analysis. *In vitro* permeation test samples were run on a C18 150 × 4.6 mm column (Kya Tech) at 1 mL min^−1^ flow rate, with a 50 μL injection volume and an oven temperature of 30°C. *In vivo* tape-stripping samples were run on a Kinetex® C18 250 × 4.6 mm column (Phenomenex) at 0.9 mL min^−1^ flow rate, with a 10 μL injection volume at 40°C. Runtime was 8.5 min and the lidocaine retention time was ~ 6.5 min. For both *in vitro* and *in vivo* experiments, calibration curves between 10 and 0.16 μg mL^−1^ with *R*^2^ = 0.999 were produced. LOD and LOQ were 0.13 μg mL^−1^ and 0.38 μg mL^−1^, respectively, *in vitro*, and 0.08 μg mL^−1^ and 0.24 μg mL^−1^, *in vivo*. The *in vivo* values correspond to 0.16 μg cm^−2^ and 0.48 μg cm^−2^ of drug per group of tape-strips.

A few detectable observations for nicotine (*in vivo*) and lidocaine (*in vitro*) were below the LOQ; these were assigned the value zero. The maximum possible effects of this choice on the *in vivo* nicotine results (calculated by comparing with results that assigned the LOQ value to all determinations less than the LOQ) were 2.5% at most for the drug amount in an individual volunteer and less than 1% for the mean values of drug mass, β and input rates. The same analysis of the *in vitro* lidocaine results showed no discernible difference in the results reported.

#### Statistics

All statistical analyses were performed with GraphPad Prism 5.01 (GraphPad Software, Inc., La Jolla, CA, USA) with *p* < 0.05 as level of significance. In the *in vivo* study, the geometric mean of the duplicate measurements in each subject were calculated, and averages for all subjects are reported as the geometric mean and 90% confidence intervals. The specific statistical tests for comparison of experimental observations are identified in the respective ‘Results’ section that follows.

## RESULTS AND DISCUSSION

**Fig. 2:**
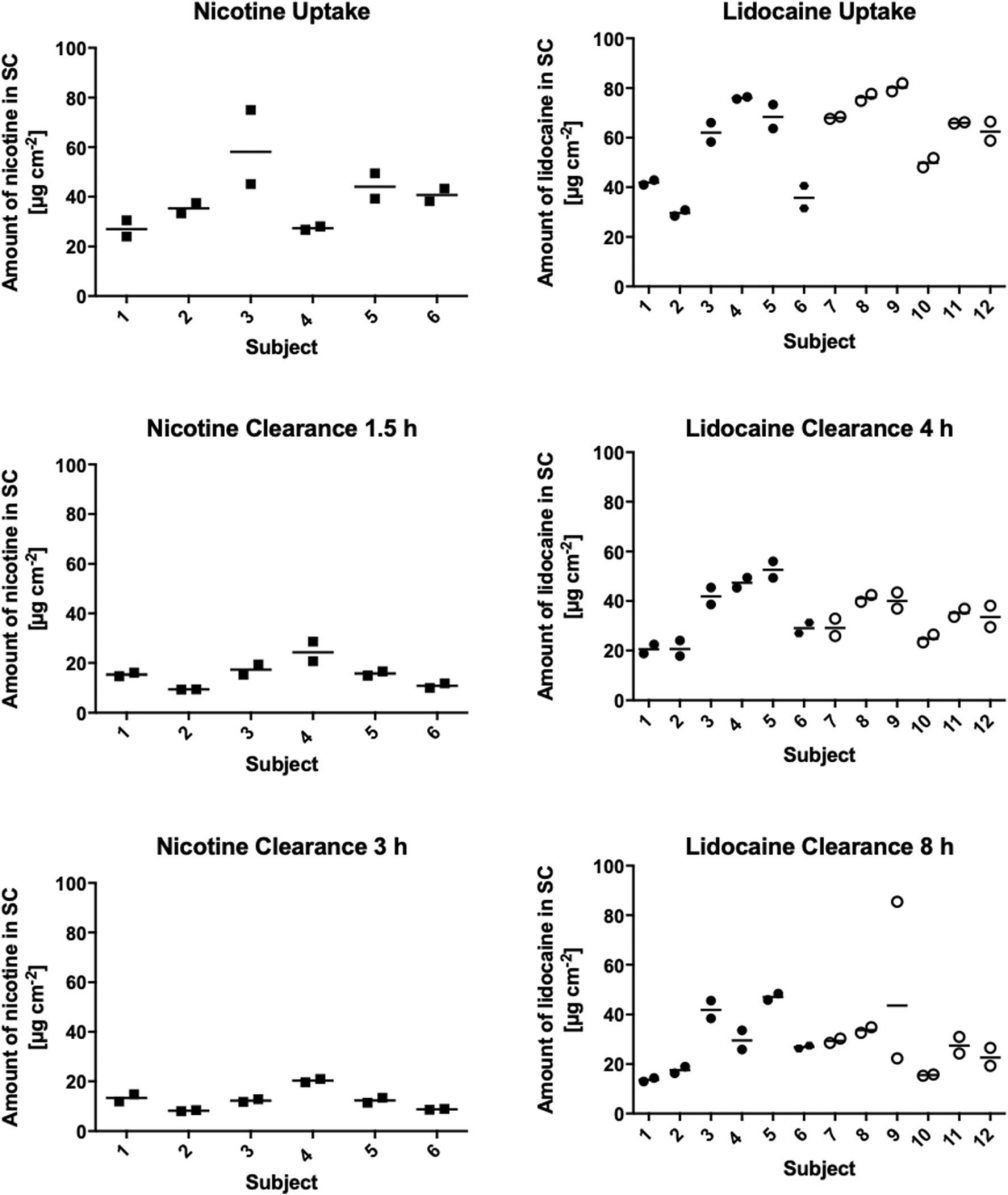
Amounts of nicotine and lidocaine in the SC after uptake and clearance; duplicate measurements and the geometric mean of these values are shown (data from the patches, subjects 1–6, are indicated by solid symbols, those from lidocaine cream, subjects 7–12, by open symbols)

The duplicated measurements of nicotine and lidocaine levels in the SC of each subject, in uptake and clearance for the three delivery systems studied, are summarised in Fig. 2; the complete dataset comprising the drug profiles as a function of SC depth is in Fig. S1 (Supplementary Information). The reproducibility of the approach was good with only two statistically significant outliers identified (the higher values for nicotine subject 3 in uptake and lidocaine subject 9 in clearance at 8 h; Grubb’s test, *p* < 0.05 (26)).

Table I summarises the information in Fig. 2 and shows—as expected—that, once the delivery system is removed, the drug is progressively cleared from the skin. The amount of drug in the SC after the first interval of clearance was reduced significantly (*p* < 0.05) for nicotine and the lidocaine cream, and was smaller, but not significantly (*p* = 0.075) for the lidocaine patch. However, the further decrease in the drug level in the SC after the second clearance interval was not significant (*p* > 0.05, repeated measures one-way ANOVA with post hoc Bonferroni test); Fig. S2. When comparing the two lidocaine products, there was no significant difference in drug amounts in the SC either following uptake or after both of the two clearance periods (repeated measures one-way ANOVA with post hoc Bonferroni test).

**Table I:** Amounts of Nicotine and Lidocaine in the SC after Uptake and Clearance; Geometric Mean (Lower–Upper 90% Confidence Interval) of the Geometric Means of Duplicate Measurements from 6 Subjects per Treatment

Amount of drug in the SC (μg cm^−2^)					
Nicotine patch		Lidocaine patch		Lidocaine cream	
2-h uptake	37.0^a^ (29.1–47.0)	12-h uptake	49.2 (35.9–67.6)	1-h uptake	66.3 (57.8–76.2)
1.5-h clearance	14.8 (11.2–19.5)	4-h clearance	33.0 (23.4–46.4)	4-h clearance	33.4 (28.5–39.1)
3-h clearance	12.0 (9.2–15.6)	8-h clearance	26.9 (18.1–39.9)	8-h clearance	27.2^b^ (20.3–36.5)

^a^35.5 (29.4–42.8) if outlier for subject 3 excluded

^b^24.4 (19.4–30.5) if outlier for subject 9 excluded

**Fig. 3:**
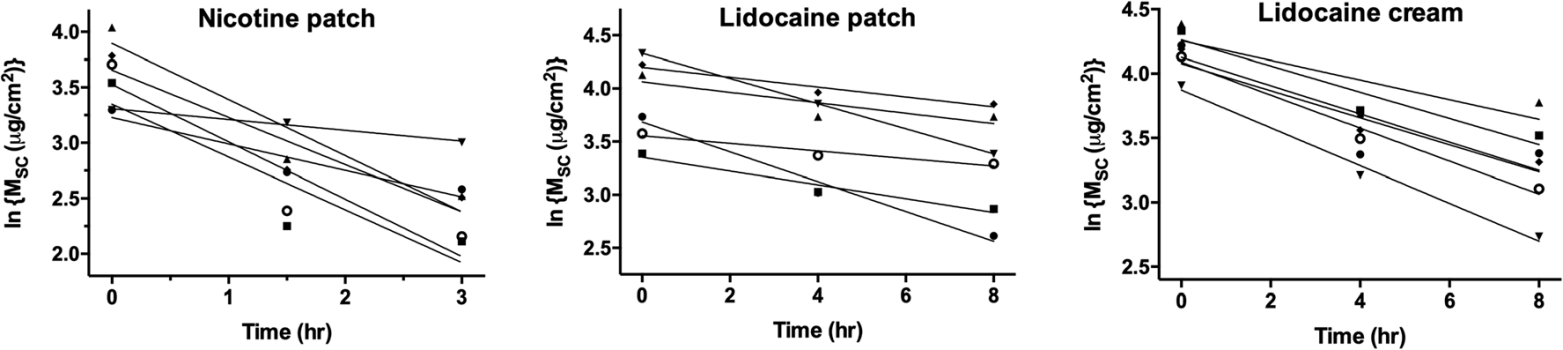
Drug clearance from the stratum corneum (SC) plotted according to Eq. 3 after removal of the applied formulation. Values of β and M_UP_ were deduced from the slope and y-axis intercept at *t* = 0 of the linear regressions indicated (each line and symbol reflecting the data from the 6 subjects studied for each formulation)

The measured drug amounts in the SC after uptake and clearance periods (Table I) were then analysed by fitting the results for each subject to Eq. 3 (Fig. 3) and by deriving slope and intercept values corresponding to the first-order elimination rate constant (β) of drug from the SC and the theoretical quantity of drug in the SC at the moment the uptake stopped (M_UP_). The average values of these metrics are presented in Table II. The linear regressions of the data were generally good with *r*^2^ values across the six subjects studied for each drug/delivery system falling between 0.89 and 1.00 except for one subject in each of the nicotine and lidocaine patch studies (*r*^2^ = 0.82 and 0.75, respectively) and two subjects in the lidocaine cream study (*r*^2^ = 0.74 and 0.65). The ‘predicted’ M_UP_ values were of course very similar to the experimental measurements reported in Table I.

**Table II:** Dermal Pharmacokinetic Metrics Deduced from the Amounts of Drug Measured in the SC after Uptake and Clearance (Values Shown Are mean ± *S*D; *n* = 6)

Dermal pharmacokinetic metric	Nicotine patch^a^	Lidocaine patch	Lidocaine cream^b^
M_UP_ ‘predicted’ (μg cm^−2^)^c^	33.8 ± 9.0	50.7 ± 19.0	61.7 ± 8.5
β (h^−1^)^d^	0.37 ± 0.17	0.076 ± 0.043	0.111 ± 0.024
*R*_1_ = β × M_UP_ (μg cm^−2^ h^−^)^e^	13.5 ± 8.1	3.9 ± 2.9	6.7 ± 0.8
β_CL1_ (h^−1^)^f^	0.61 ± 0.32	0.100 ± 0.045	0.172 ± 0.021
*R*_1,CL1_ = β × M_UP_ (μg cm^−2^ h^−1^)^g^	25.5 ± 16.2	5.3 ± 2.8	11.5 ± 2.3
*R*_2_ = (M_UP_ − M_CL_)/∆t (μg cm^−2^ h^−1^)^h^	15.2 ± 8.9	4.2 ± 2.1	8.3 ± 1.5

^a^If the outlier is excluded, M_UP_ ‘predicted’ = 32.3 ± 6.2, β = 0.36 ± 0.16, *R*_1_ = 12.2 ± 6.3; β_CL1_ = 0.58 ± 0.31, *R*_1,CL1_ = 22.5 ± 13.3, and *R*_2_ = 13.8 ± 7.3

^b^If the outlier is excluded, M_UP_ ‘predicted’ = 63.1 ± 10.7, β = 0.125 ± 0.024, *R*_1_ = 7.9 ± 2.4; β_CL1_, *R*_1,CL1_ and *R*_2_ are unchanged

^c^Drug amount predicted in the SC at the end of the uptake period from the intercept of the linear regressions in Fig. 3 according to Eq. 3

^d^First-order clearance rate constant of drug from the SC calculated from the slope of the linear regressions in Fig. 3 according to Eq. 3

^e^Input rate of the drug from the SC into the viable skin at the end of the uptake period according to Eq. 4 if β is constant for the entire clearance interval

^f^First-order clearance rate constant of drug from the SC for the shorter clearance time calculated according to Eq. 5

^g^Input rate of the drug from the SC into the viable skin at the end of the uptake period estimated from the shorter clearance time and the geometric mean value for M_UP_ according to Eq. 5

^h^Input rate of the drug from the SC into the viable skin estimated from the difference between drug amounts in SC after uptake and after a period (∆t) of clearance according to Eq. 6

The derived first-order elimination rate constants (β) of drug from the SC, although relatively consistent, revealed a degree of inter-subject variability not uncommon for human, *in vivo* data associated with skin permeation. A one-way analysis of variance (ANOVA) of the β results for the three delivery systems studied, followed by Tukey’s multiple comparison test, revealed that the rate constant measured for nicotine was significantly greater than those determined for lidocaine, whether applied as a patch (*p* < 0.001) or as a cream (*p* < 0.01). In contrast, there was no significant difference in the β values for lidocaine when administered as a patch or as a cream. A more detailed discussion and analysis of these results are provided in the Supplementary Information.

The fitted values of M_UP_ and β were then used, in accord with Eq. 4, to estimate the input rate (*R*_1_) of the drugs from the SC into the viable epidermis. These results, along with those for the rate constant and input rate derived for the shorter clearance interval (β_CL1_ and *R*_1,CL1_, respectively) are presented in Table II. Values of *R*_2_ were also assessed (see Table II) directly from the experimental measurements of drug in the SC following uptake and after the shorter clearance period, as indicated in Eq. 5 (6–9). It is worth emphasising that, when using the tape-stripping method, drug clearance from the SC is measured after the drug formulation has been removed from the skin surface. To estimate as closely as possible the input rate while the formulation is on the skin surface, the clearance rate should be measured as soon as possible after the drug product has been removed with the constraint that the clearance period must be long enough so that the drug amount in the SC decreases by a statistically significant amount compared to the amount after uptake.

It is immediately apparent that there is a close overlap between the values of *R*_1_ and *R*_2_ for each of the three drug formulations, suggesting that the two approaches are viable methods to provide a metric related to the rate at which a topically applied drug is able to reach the ‘compartment’ in which many targets for the treatment of dermatological disease are found. Statistically speaking (again based on a one-way ANOVA followed by Tukey’s multiple comparison test), for both *R*_1_ and *R*_2_, the input rate of nicotine from the transdermal patch was significantly greater than that of lidocaine from its patch product; on the other hand, there was no difference between the values of either *R*_1_ or *R*_2_ when comparing the nicotine patch to lidocaine cream, or when comparing the lidocaine patch and cream formulations.

It is entirely plausible that a more mechanistic diffusion modelling approach may permit alternative strategies for the interpretation of the measurements presented here (as has been shown previously [7]). However, given the complexities of drug delivery from a topical dermatological formulation (including metamorphosis of the formulation and the impact of this transformation on drug delivery), in addition to the variability inherent to the quantitation of percutaneous absorption, a simpler measurement/analysis strategy that is informed by a knowledge of the diffusion process is better justified. This study must be viewed as an initial proof-of-concept, therefore, not the final word, and further work is required to explore these issues in the appropriate depth.

**Fig. 4:**
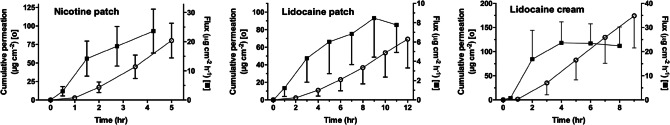
*In vitro* skin permeation results for the formulations considered. Data (mean *± S*D; *n* = 6 for nicotine and *n* = 7 for lidocaine) are presented as the cumulative amount of drug absorbed (open symbols, left axis) and as the flux, plotted at the mid-point of the sampling interval, as a function of time (closed symbols, right axis)

The *in vitro* skin permeation results are summarised in Fig. 4, where each panel provides both the cumulative penetration and the flux of the drug into the receptor compartment of the diffusion cell. After 5 h, the average nicotine flux across the skin from the Nicotinell® patch over the final sampling interval (3.5 to 5 h) was 23.7 (± 7.5) μg cm^−2^ h^−1^. Given that the 10 cm^2^ system is labelled to deliver 7 mg over 24 h, the expected *in vivo* performance of the patch corresponds to a flux of 29 μg cm^−2^ h^−1^, and suggests a reasonable *in vitro*-*in vivo* correlation, therefore. The *in vitro* flux results are also close to the *in vivo* input rate for the first clearance interval *R*_1,CL1_ (25.5 ± 16.2) μg cm^−2^ h^−1^ and within a factor of 2 of the *in vivo* values of *R*_1_ and *R*_2_ for nicotine discussed above (13.5 ± 8.1 and 15.2 ± 8.9 μg cm^−2^ h^−1^, respectively) which were determined following only 2 h of patch wear. The average *in vitro* flux deduced over the final hour of a 2-h application was 14.2 (± 6.0) μg cm^−2^ h^−1^. The Summary of Product Characteristics (SmPC) for this matrix system (27) states that, ‘following a single application… there is an initial 1-2 hour delay followed by a progressive rise in nicotine plasma concentrations, with a plateau attained at about 8-10 hours after application’. While it is true that the rate of drug release from a matrix system will initially be fast and then slow down over time, the presence of the rate-limiting SC causes the delivery into the patient (or into an IVPT receptor phase, or into the viable skin) to follow the profile described in the product’s SmPC. For that reason, which is well-understood in the field, the comparison of the nicotine input rate, derived from SC sampling, with the labelled, average flux of 7 mg per 24 h is reasonable.

The Summary of Product Characteristics (SmPC) of the Versatis® medicated plaster used (28) indicates that the 140 cm^2^ patch contains a total of 700 mg of drug, corresponding to a loading of 5 mg cm^−2^. The SmPC also highlights that the plaster, when worn for the recommended 12 h, delivers systemically ‘about 3 ± 2 % of the total applied lidocaine dose’, i.e. the equivalent of 150 (± 100) μg cm^−2^, at an average flux of 12.5 (±8.3) μg cm^−2^ h^−1^. The *in vitro* results in Fig. 4 agree quite well with this information: cumulative permeation in 12 h was nearly 70 μg cm^−2^ and the terminal flux when the patch was removed was ~ 8 μg cm^−2^ h^−1^. The *in vivo* input rate (whether *R*_1_ or *R*_2_) deduced from the SC sampling experiment (Table II) was about half (and modestly larger for *R*_1,CL1_) that measured *in vitro* but still within the range of values reported in the SmPC. It is important to point out that the sample sizes for both the *in vivo* and *in vitro* experiments are small, reducing the power of the comparisons with the reported patch results; better agreement might be observed with larger sample sizes.

Lidocaine uptake into the SC *in vivo* from the commercial cream following a 1-h application was about 70 μg cm^−2^ (Table I) and the deduced input rate to the viable epidermis was similar for *R*_1_ (6.7 ± 0.8 μg cm^−2^ h^−1^) and *R*_2_ (8.3 ± 1.5 μg cm^−2^ h^−1^) and a little larger for the first clearance period (11.5 ± 2.3 μg cm^−2^ h^−1^). The *in vitro* skin penetration experiments reinforced the apparent rapid uptake of the drug into the skin compared to the lidocaine patch, with lag times, respectively, of approximately 1.5 and 4 h, a fact quite possibly attributable to the significant presence of two excipients in the formulation, specifically propylene glycol (at 7.5% w/w) and benzyl alcohol (1.5% w/w). This was also evident from the spatial distribution of lidocaine in the SC as shown in Fig. S1 (Supplementary Information); while the drug was fairly evenly distributed after patch wear, the short duration of cream application resulted in an initially high loading and a steep concentration gradient. It seems reasonable to speculate, upon application of the cream to the skin, that the transformation, or ‘metamorphosis’, of the formulation may lead to a rapid input of drug into the SC as these small molecular weight excipients themselves are taken up and/or lost by evaporation, thereby both (potentially) acting as penetration enhancers and increasing the drug’s thermodynamic activity in the evolving residual phase remaining on the skin surface. Notably, lidocaine flux from the cream reached a maximum at about 3 h that was maintained until the end of the 9-h experiment. Given the relatively large dose of cream applied, it is reasonable to anticipate that a steady flux of the drug would have been sustained for a further period of time.

As a more general point with respect to the lidocaine cream data, it is strongly recommended that estimates of drug concentration in the target tissue from a topical dermatological formulation should be measured from doses that are relevant to their intended use. While acknowledging that the lidocaine cream dose is high relative to that of other topical products, it is worth noting that problems with dose precision and/or analytical sensitivity have not been experienced when using formulations that are applied in much smaller quantities. For example, a study that measured econazole in tape-stripped SC involved dosing with 4.5 mg/cm^2^ of 1% econazole nitrate creams (7); similarly, a diclofenac investigation (8) used the recommended doses of the formulations studied (i.e. either 10 or 20 mg/cm^2^).

It should also be emphasised that the determination of drug input rate to the viable tissue using skin sampling by tape-stripping does not require measurement of SC mass collected. The protocol is designed, by use of the TEWL measurements, to collect most of the SC (and thereby most of the drug in the SC), which reduces the site-to-site variability (and also minimises investigator-to-investigator variability in studies with multiple investigators). In this study, the SC masses collected on the tape-strips from the three products and three measurement times were consistent (Table S1) and showed no statistically significant differences (*p* < 0.05). Overall, about 75% of the SC thickness was removed by the tape-strips, corresponding to approximately 90% of the drug (see Supplementary Information).

Finally, as recently reported for acyclovir (6), the experimentally determined ‘input’ fluxes from the SC sampling experiments reported here can be equated with the product of the free drug concentration (C*) at the site of action (the sub-SC ‘compartment’, exemplified by the basal epidermis) and a heterogeneous rate constant (P_D_), describing drug clearance from the site (12–16); P_D_ = D_D_/h_D_, where D_D_ is the drug’s diffusivity in the dermis, and is calculable from molecular weight with available algorithms (10), and h_D_ is the distance that a drug must diffuse from the basal epidermis to the microcirculation where it is cleared from the skin. In this way, it is possible to estimate values of C* achieved for nicotine and lidocaine, as shown in Table III.

**Table III:** Estimation of Drug Concentrations at the Site of Action in the Viable Skin (C*) from SC Sampling Results for Nicotine Delivered from a Patch and for Lidocaine Delivered from a Medicated Plaster and from a Cream

Drug (delivery system)	(M_UP_ − M_CL_)/∆t (μg cm^−2^ h^−1^)^a^	D_D_ (cm^2^h^−1^)^b^	P_D_ (cm h^−1^)^c^	C* (μg cm^−3^)
Nicotine (patch)	15.2	0.0101	1.015	15.0
Lidocaine (plaster)	4.2	0.0076	0.757	5.6
Lidocaine (cream)	8.3	0.0076	0.757	11.0

^a^Average input flux from Table II (defined as *R*_2_) determined from SC sampling experiments

^b^Average value from two algorithms proposed by Krestos *et al*. (10)

^c^P_D_ = D_D_/h_D_, where h_D_ is assumed to be 100 μm (6)

It is perhaps unsurprising that the estimations of C* for nicotine and lidocaine, which may be considered as relatively good skin penetrants, are considerably larger than that assessed for the poorly absorbed acyclovir (~ 0.04 μg cm^−3^) (6).

## CONCLUSIONS

The research described in this paper aimed to further address the challenge of assessing the topical bioavailability of a drug at its site of action in the skin. Specifically, experiments were designed to test the hypothesis that interrogation of drug levels in the stratum corneum (SC) *in vivo* can permit the ‘input rate’ into the underlying, living skin ‘compartment’ (where many dermatological disease targets are found) to be deduced. The results demonstrate that the interpretation of drug amounts in the SC after periods of uptake, when the formulation is in contact with the skin, and after periods of ‘clearance’, post-removal of the formulation, yield deduced ‘input rates’ that are consistent with known product performance and in broad agreement with conventional *in vitro* skin permeation test measurements. This further validation of the SC sampling methodology provides additional evidence to support its application to the assessment of local, topical bioavailability of drugs that act within the skin. The *in vi*vo nature of the measurements suggests, furthermore, that the approach may find useful application in the determination of topical drug product bioequivalence for which an approach (or a ‘tool-kit’ of approaches, which might include more specialised microdialysis and spectroscopic techniques, for example) to avoid the need for clinical end-point studies is an important current goal.

## Supplementary Information

ESM 1 (DOCX 2.79 MB)
