# Supplementary material for: Assessment of Drug Delivery Kinetics to Epidermal Targets In Vivo
Source: AAPS J. 2021 Mar 29;23(3):49. doi: 10.1208/s12248-021-00571-3 (PMC8007522; doi:10.1208/s12248-021-00571-3)
Supplement: Supplementary file 1 — (DOCX 2.79 MB) [file 12248_2021_571_MOESM1_ESM.docx]

**Assessment of drug delivery kinetics to epidermal targets *in vivo***

**M. Hoppel^1^, M.A.M. Tabosa^1^, A.L. Bunge^2^, M.B. Delgado-Charro^1^, R.H. Guy^1,3^**

^1^University of Bath, Department of Pharmacy & Pharmacology, Claverton Down, Bath, BA2 4LZ, UK

^2^Department of Chemical Engineering, Colorado School of Mines, Golden, CO 80401, USA

^3^Correspondence: Prof. R.H. Guy, University of Bath, Department of Pharmacy & Pharmacology, Claverton Down, Bath, BA2 4LZ, UK. [r.h.guy@bath.ac.uk](mailto:r.h.guy@bath.ac.uk); tel. +44-(0)1225-384901

*Running head:* Drug delivery to epidermal targets

**Supplementary information**

*Figure S1: Profiles of nicotine and lidocaine concentrations plotted as a function of depth into the SC after uptake and two clearance periods. The individual, duplicate measurements made in 6 subjects are shown on each profile (a few data points are out of range and not shown). The patch uptake period was 12 hours; for the cream, uptake after 1 hour of application was assessed.*


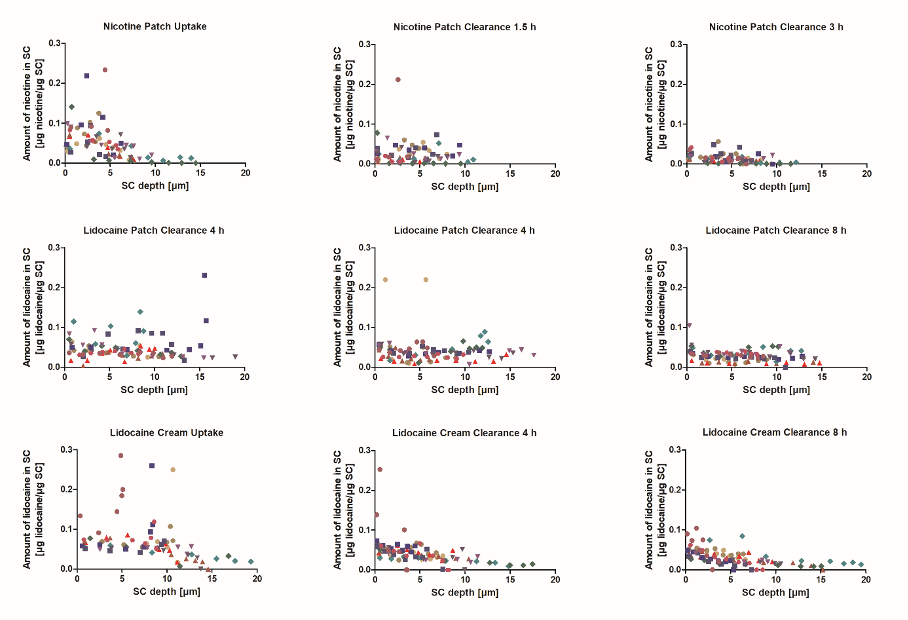


In the above plots, drug concentration is plotted at the centre of the SC thickness of the last strip; i.e., the depth of the n^th^ tape strip (z_n_) is calculated from the SC mass per area on the j^th^ tape (M_SC,j_/A)

$$z_{n}=\left[\sum_{j=1}^{n-1} \frac{M_{SC,j}}{A}+\frac{M_{SC,n}}{2A}\right]/\rho_{SC}$$

where ρ_SC_ is the density of the SC.

The tape stripping protocol is designed to collect (and measure) most of the drug in the SC by collecting a large fraction of the SC. For drug concentrations at steady state (i.e., the concentration profile decreases linearly to approximately zero with depth into the SC), 75% of the drug is collected if half of the SC is stripped; approximately 90% of the drug is collected if 75% of the SC is stripped. If most of the drug is collected, then variations in the exact amount of SC stripped contribute minimally to the measurement variability. In this study, the SC masses collected on the tape strips (Table S1) were consistent between products and measurement time; there was no statistically significant differences (p < 0.05). Overall, approximately 10 μm of SC was removed by the tape strips, which corresponds to roughly three-quarters of the typical SC thickness (11.7 ± 3.2 µm) on the forearms of volunteers with healthy skin [1].

*Table S1. SC mass collected on the tape strips for both drugs and the three delivery systems*

| **SC mass collected (mg cm^-2^)^a^** | | | | | |
| --- | --- | --- | --- | --- | --- |
| **Nicotine patch** | | **Lidocaine patch** | | **Lidocaine cream** | |
| 2 hr uptake | 0.76  (0.55-1.04) | 12 hr uptake | 1.27  (1.05-1.54) | 1 hr uptake | 1.19  (0.85-1.66) |
| 1.5 hr clearance | 0.74  (0.60-0.91) | 4 hr clearance | 1.08  (0.84-1.39) | 4 hr clearance | 0.88  (0.63-1.23) |
| 3 hr clearance | 0.87  (0.74-1.01) | 8 hr clearance | 1.11  (0.89-1.39) | 8 hr clearance | 0.92  (0.64-1.31) |

^a^ The thickness of the SC removed by the tape strips (in μm) is 10 × the SC mass collected (in mg/cm^2^) assuming the density of the SC is approximately 1 g/cm^3^.

Figure S2 shows the clearance-to-uptake ratios representing the fraction of the amount in the SC at uptake that remains in the SC after the first and second intervals of clearance. For both drugs and each formulation, this ratio is statistically significantly different from no clearance (i.e., the ratio is one) but, for the first and second clearance intervals, the values are not statistically significant different.


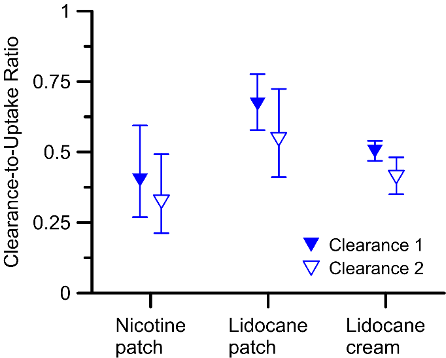


*Figure S2. Clearance-to-uptake ratios for clearance 1 and clearance 2 for the nicotine and lidocaine patch and lidocaine cream.*

[1] Herkenne C, Naik A, Kalia YN, Hadgraft J, Guy RH. Pig ear skin ex vivo as a model for in vivo dermatopharmacokinetic studies in man. Pharm Res. 2006; 23: 1850-1856.

Validity of the assumption inherent the analysis of clearance kinetics

If the SC behaves approximately as a pseudo-homogeneous membrane with constant values for diffusion and partitioning and with no other significant reactions (e.g., reversible protein binding), then it has been shown that the ratio of the drug amount in such a membrane after a period of clearance compared with uptake is “monoexponential” (i.e., the log of the clearance-to-uptake ratio varies linearly with time) - see Figure 6 in N’Dri-Stempfer et al. (2009), i.e., ref. 7 in the present paper. If the uptake time is smaller than 3 x t_lag_ (i.e., approximately the time to reach steady state if the concentration of the applied dose does not change too much), then there will be a slight shift in the time before the slope becomes monoexponential. That shift is quite small unless the uptake time (*t_o_*) is smaller than about 0.6 x t_lag_. For nicotine, the *in vitro* lag time was about 2 h; for lidocaine, the *in vitro* lag times were about 1 h and 4 h from the cream and patch, respectively. Of course, the lag time *in vitro* will be longer than that *in vivo* because a functioning microcirculation is absent in the thick (> 700 μm) excised porcine skin used. Nevertheless, using these IVPT estimates of lag time, we can expect that the *in vivo* uptake times (t_o_) were larger than 0.6 x t_lag_ for all 3 products: 2 h uptake compared with 0.6 x t_lag_ = 1.2 h for nicotine, 1 h uptake compared with 0.6 x t_lag_ = 0.6 h for the lidocaine cream, and 12 h uptake compared with 0.6 x t_lag_ = 2.4 h for the lidocaine patch.

By measuring two clearance points instead of just one, we determined that for these three products the effective first-order clearance rate constants do decrease in time. Thus, for comparison with the approximately steady-state *in vitro* flux and the labelled delivery rates from the patches, the effective first-order rate constant derived for the shorter clearance interval and the measured uptake amount may provide a reasonable estimate for the input rate from the formulation and this has been demonstrated experimentally in this study.

The cause(s) for the decreasing rate constants are not known but possible explanations include changes in hydration (e.g., after an occlusive patch is removed), clearance of putative permeation enhancers (e.g., propylene glycol), drug loss from the SC by, for example, evaporation, protein binding, drug ionization and entrapment in corneocytes, and others as yet unidentified. Comments on these phenomena have been presented in the text of the paper and we intend to address this subject in subsequent work.
